# Influence of spacer chain lengths and polar terminal groups on the mesomorphic properties of tethered 5-phenylpyrimidines

**DOI:** 10.3762/bjoc.5.63

**Published:** 2009-11-09

**Authors:** Gundula F Starkulla, Elisabeth Kapatsina, Angelika Baro, Frank Giesselmann, Stefan Tussetschläger, Martin Kaller, Sabine Laschat

**Affiliations:** 1 Institut für Organische Chemie, Universität Stuttgart, Pfaffenwaldring 55, 70569 Stuttgart, Germany; 2 Institut für Physikalische Chemie, Universität Stuttgart, Pfaffenwaldring 55, 70569 Stuttgart, Germany

**Keywords:** calamitic, liquid crystals, 5-phenylpyrimidines

## Abstract

Based on 5-(4-hydroxyphenyl)-2-octylpyrimidine **8**, 5-phenylpyrimidine derivatives **3**–**7**, **9** with different spacer chain lengths (C_2_ up to C_6_) and different terminal polar groups (Br, Cl, N_3_, OH, CN) were synthesized by etherification and nucleophilic substitution. The mesomorphic behaviour of these compounds was investigated by differential scanning calorimetry (DSC), polarizing optical microscopy (POM) and X-ray diffraction (WAXS and SAXS) and revealed smectic A mesophases for bromides, chlorides and azides **3**, **4** and **6**. For these compounds a maximum phase width was observed for the C_5_ spacer regardless of the terminal group, whereas the hydroxy- and cyano-substituted derivatives **5** and **7**, respectively, were non mesomorphic and showed only melting transitions.

## Introduction

**Scheme 1:**
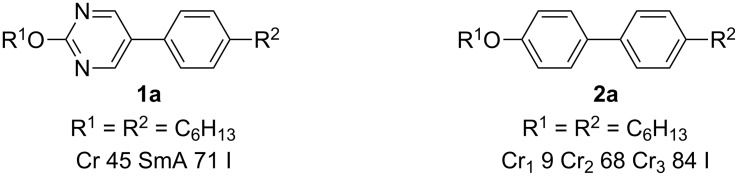
Comparison of mesomorphic properties of **1a** and **2a**.

A tremendous amount of work has been done on calamitic liquid crystals, which has led to applications in the field of LC displays [1]. Among the large family of various calamitic mesogens 2-alkoxy-5-phenylpyrimidines **1** are prominent members due to the fact that the two nitrogen atoms increase the polarity of the rigid rod core structure (Scheme 1) as exemplified by the derivative **1a** which displays a SmA phase between 45 °C and 71 °C, while the corresponding biphenyl derivative **2a** with the same terminal alkyl chains does not have any liquid crystallinity [1].

Whereas three regioisomeric phenylpyrimidines are possible, i.e. 4-, 5-, and 2-phenylpyrimidine, only the latter two are suitable for liquid crystals. Furthermore 5- and 2-phenylpyrimidines differ in their overall conformation. According to ab initio calculation by Barone [2], 2-phenylpyrimidine is almost planar, whereas 5-phenylpyrimidine has a twisted conformation with a dihedral angle of 43.1° [3]. The different conformations together with differences of the polarisation and dipole moment between 2- and 5-phenylpyrimidines also lead to different mesomorphic properties as was shown by Lemieux for phenylpyrimidines tethered to terminal trisiloxanes [4–5] and by Tschierske for dimeric phenylpyrimidines tethered to oligoethyleneglycol units [6].

**Scheme 2:**
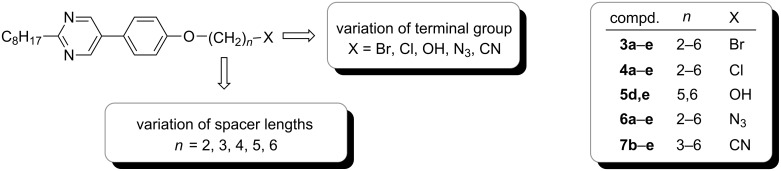
Variation of spacer lengths and terminal group at 5-phenylpyrimidine.

We recently reported the synthesis of 1,1′-biisoquinolines tethered to calamitic subunits [7]. During these studies we discovered that the 5-phenylpyrimidine building block **3e** already displayed a SmA mesophase. We thus wondered whether variation of the spacer chain lengths and terminal group X (Scheme 2) might have significant influence on the mesomorphism. The results of this study are discussed below.

## Results and Discussion

**Scheme 3:**
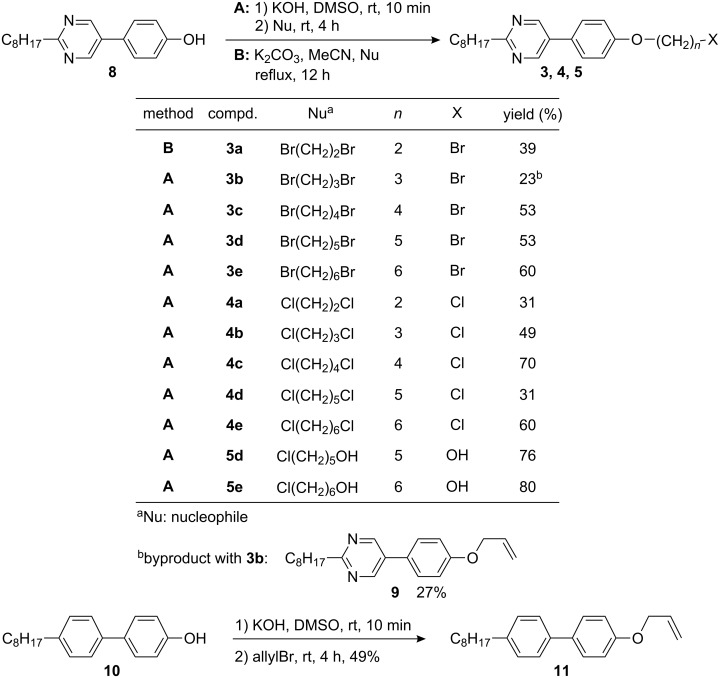
Synthesis of compounds **3**–**5**, **9** and **11**.

**Syntheses:** In order to obtain different series **3**–**7** the known 5-(4-hydroxyphenyl)-2-octylpyrimidine **8** [7–12] was used as starting material (Scheme 3).

**Scheme 4:**
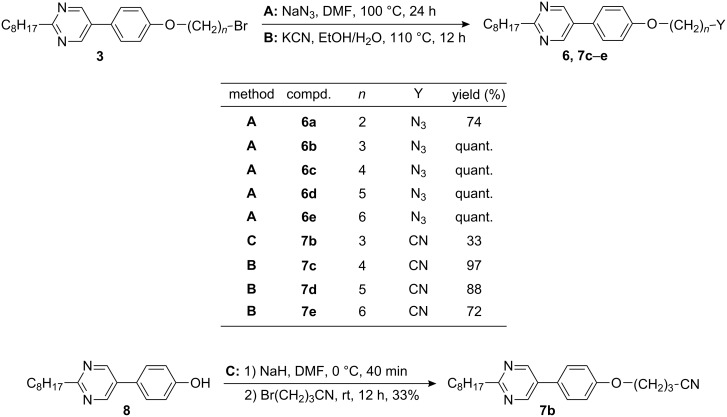
Synthesis of compounds **6** and **7**.

Compound **8** was deprotonated with KOH in DMSO at room temperature for 10 min followed by addition of 1,ω-dibromoalkane. After 4 h, the reaction mixtures were purified and the desired bromides **3b**–**e** were isolated in 23 up to 60% yield. When 1,3-dibromopropane was used, 27% of the elimination product **9** was isolated as byproduct. For comparison the corresponding 4-allyloxy-4′-octylbiphenyl **11** was prepared in 49% yield by allylation of 4-hydroxy-4′-octylbiphenyl. Compound **3a** was obtained by etherification of 5-(4-hydroxyphenyl)-2-octylpyrimidine **8** using K_2_CO_3_ in MeCN under reflux for 12 h to yield 39%. The synthesis of series **4** with chloride as terminal group proceeded in a similar way by using 1,ω-dichloroalkanes giving **4a**–**e** in 31–70% yield. Upon deprotonation of **8** under the conditions described above, followed by treatment with 5-bromopentanol or 6-bromohexanol, the hydroxy compounds **5d** and **5e** were isolated in 76% and 80% yield, respectively. To obtain the azides **6**, bromides **3a**–**e** were treated with NaN_3_ in DMF at 100 °C for 24 h and the products **6a**–**e** were isolated in 74% up to quantitative yield (Scheme 4). In a similar manner, the cyanides **7** were prepared from the bromides **3**. Treatment of the bromides **3c**–**e** with KCN in EtOH/H_2_O at 110 °C for 12 h leads to the cyanides **7c**–**e** in 72 to 97% yield.

Since the yields of **3b** were limited due to side reactions like elimination, the respective cyanide **7b** was prepared by deprotonation of hydroxy derivative **8** with NaH in DMF for 40 min at 0 °C followed by addition of 1-bromo-3-propionitrile at room temperature. After 12 h, the cyanide **7b** was obtained in 33% yield.

**Mesomorphic properties:** Mesomorphic properties of compounds **3**–**10** were investigated by differential scanning calorimetry (DSC), polarizing optical microscopy (POM) and X-ray diffraction (WAXS and SAXS). The DSC results for bromides **3** are summarized in Table 1.

**Table 1:** Phase transition temperatures [°C] and enthalpies [kJ/mol] of compounds **3**.^a^

**3**	*n*	Cr_1_	*T*	Δ*H*	Cr_2_	*T*	Δ*H*	SmA	*T*	Δ*H*	I	

**a**	2	•	52	3.9	•	63	32.2	-	-	-	•	2. heating
		•	29	−25.8	-	-	-	•	56	−5.6	•	2. cooling
**b**	3	•	46	13.0	-	-	-	•	52	1.9	•	2. heating
		•	35	−13.9	-	-	-	•	52	−3.2	•	2. cooling
**c**	4	•	66	31.1	-	-	-	-	-	-	•	2. heating
		•	49	−24.1	-	-	-	•	63	−5.0	•	2. cooling
**d**	5	•	40	17.6	-	-	-	•	57	5.1	•	2. heating
		•	24	−16.7	-	-	-	•	60	−5.3	•	2. cooling
**e**	6	•	64	36.3	-	-	-	-	-	-	•	2. heating
		•	35	−23.9	-	-	-	•	58	−5.5	•	2. cooling

^a^Cr crystalline; SmA smectic A; I isotropic; • phase was observed; - phase was not observed. Heating and cooling rate: 10 K/min.

**Figure 1:**
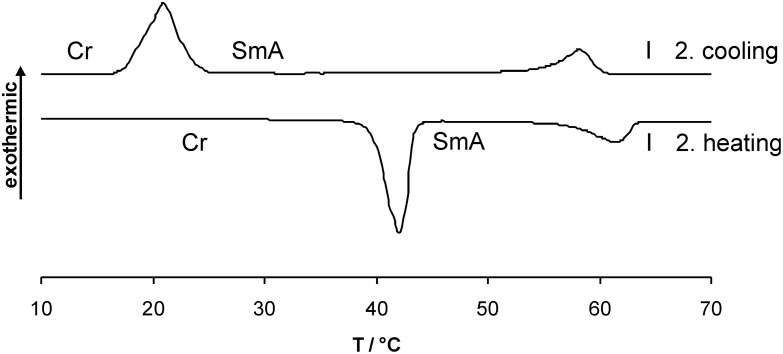
DSC curve of compound **3d** (heating/cooling rate 10 K/min).

Whereas compounds **3a,c,e** with even chain lengths of the spacer displayed monotropic SmA phases, compounds **3b,d** with odd chain lengths displayed enantiotropic SmA phases. A typical DSC curve of derivative **3d** with a pentyloxy spacer is shown in Figure 1.

On the third heating a melting transition at 40 °C into the SmA phase and a clearing transition at 57 °C could be observed. Subsequent cooling revealed an isotropic to SmA transition at 60 °C and a crystallization peak at 24 °C. The SmA to crystalline transition tends to strong supercooling in the order of 10–30 K (Table 1, Figure 1).

**Figure 2:**
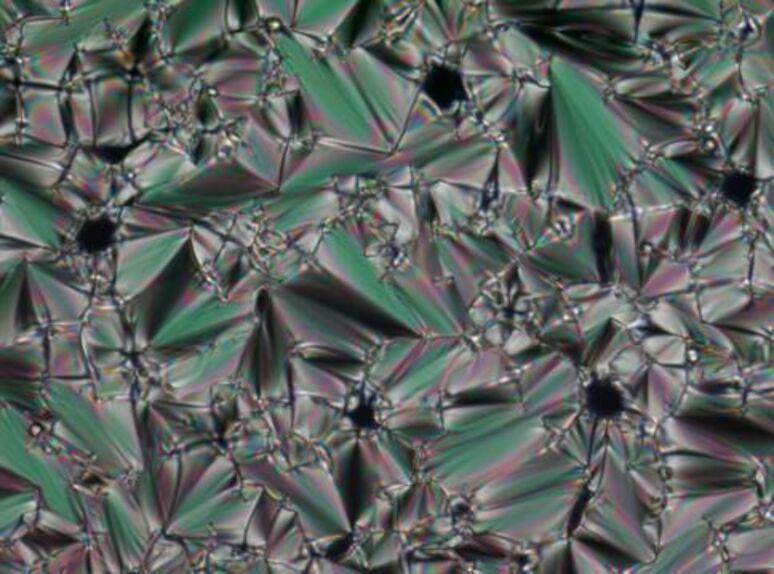
Fan-shaped texture of **3e** under crossed polarizers upon cooling from the isotropic liquid (magnification 200×): at 55 °C (cooling rate 5 K/min): smectic A phase.

POM observation of **3a**–**e** revealed fan-shaped textures typical of SmA phases. An illustrative example is depicted in Figure 2. The assignment of the SmA mesophases was further confirmed by XRD experiments (see the Supporting Information).

It should be noted that the allyloxy-substituted byproduct **9** showed a smectic mesophase between 50 °C and 67 °C as well. In contrast, the corresponding 4-allyloxy-4′-octylbiphenyl **11** showed only isotropic melting at 92 °C. The DSC results of chlorides **4** are summarized in Table 2.

**Table 2:** Phase transition temperatures [°C] and enthalpies [kJ/mol] of compounds **4**.^a^

**4**	*n*	Cr	*T*	Δ*H*	SmA	*T*	Δ*H*	I	

**a**	2	•	50	21.6	•	55	1.4	•	2. heating
		•	25	−18.7	•	53	−1.5	•	2. cooling
**b**	3	•	37	11.9	•	53	3.9	•	2. heating
		•	23	−11.8	•	58	−4.2	•	2. cooling
**c**	4	•	63	21.9	•	67	2.7	•	2. heating
		•	46	−23.5	•	70	−4.9	•	2. cooling
**d**	5	•	42	15.7	•	56	5.0	•	2. heating
		•	27	−17.0	•	62	−4.8	•	2. cooling
**e**	6	•	55	20.8	•	59	4.7	•	2. heating
		•	42	−25.3	•	64	−4.5	•	2. cooling

^a^Cr crystalline; SmA smectic A; I isotropic; • phase was observed; - phase was not observed. Heating and cooling rate: 10 K/min for **4a**–**d**, 5 K/min for **4e**.

**Figure 3:**
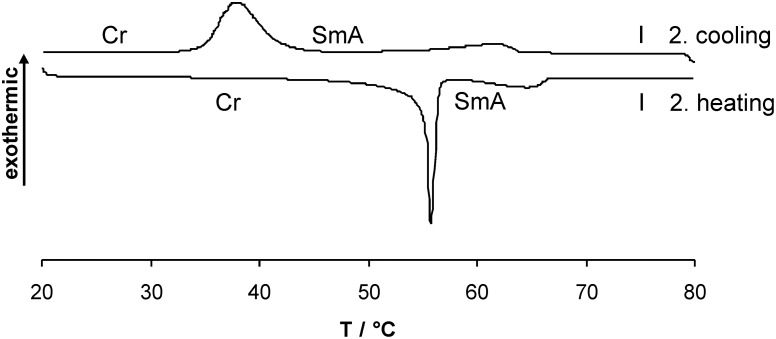
DSC curve of compound **4e** (heating/cooling rate 5 K/min).

All members **4a**–**e** showed enantiotropic SmA phases. For compounds **4a,c,e** with even numbered spacer lengths smaller mesophase widths were observed as compared to compounds **4b,d** with odd numbered spacer lengths. Furthermore an *odd–even* effect of both melting and clearing points was found. A typical DSC curve which is shown in Figure 3 for chloride **4e** with hexyloxy spacer, revealed a melting transition at 59 °C to the smectic A phase and a clearing transition at 66 °C upon a second heating. Upon the second cooling run an isotropic to SmA transition at 64 °C and a crystallization peak at 42 °C were observed.

**Figure 4:**
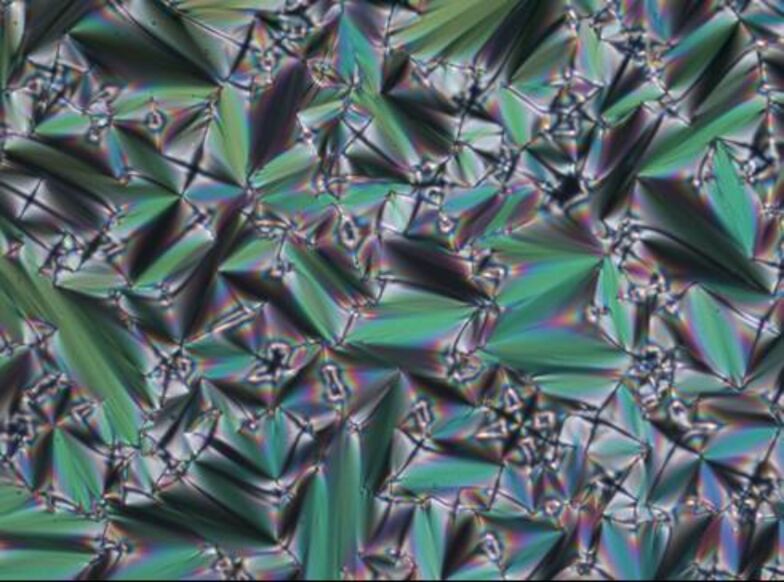
Fan-shaped texture of compound **4d** at 45 °C upon cooling from the isotropic liquid (cooling rate 1 K/min) (magnification 200×).

POM investigation displayed fan-shaped and focal conic textures, as exemplified in Figure 4. XRD experiments proved the smectic phase.

In contrast to the bromides **3** and chlorides **4**, the hydroxy and azide derivatives **5a,b** and **7b**–**e** were non mesomorphic and showed only melting transitions at 76 °C and 75 °C for compounds **5a,b** and at 77 °C, 86 °C, 67 °C and 68 °C for the azides **7b**–**e**, respectively (upon heating or cooling) in the DSC curve. Presumably, the higher polarity of the terminal hydroxy group with respect to the azido group, together with hydrogen bonding, inhibits mesophase formation. Next the azides **6** were investigated by DSC (Table 3).

**Table 3:** Phase transition temperatures [°C] and enthalpies [kJ/mol] of compounds **6**.^a^

**6**	*n*	Cr_1_	*T*	Δ*H*	Cr_2_	*T*	Δ*H*	SmA	*T*	Δ*H*	I	

**a**	2	•	48	30.6	-	-	-	-	-	-	•	2. heating
		•	44	−31.4	-	-	-	-	-	-	•	2. cooling
**b**	3	•	45	11.9	-	-	-	•	49	1.1	•	2. heating
		•	34	−13.7	-	-	-	•	47	−2.9	•	2. cooling
**c**	4	•	42	25.3	-	-	-	•	60	5.0	•	2. heating
		•	28	−17.8	-	-	-	•	63	−5.3	•	2. cooling
**d**	5	•	7	0.9	•	28	17.2	•	55	3.4	•	2. heating
		•	1	−0.6	•	11	−16.0	•	57	−4.7	•	2. cooling
**e**	6	•	41	26.5	-	-	-	•	53	6.1	•	2. heating
		•	20	−22.2	•	25	−0.5	•	58	−5.9	•	2. cooling

^a^Cr crystalline; SmA smectic A; I isotropic; • phase was observed; - phase was not observed. Heating and cooling rate: 10 K/min.

**Figure 5:**
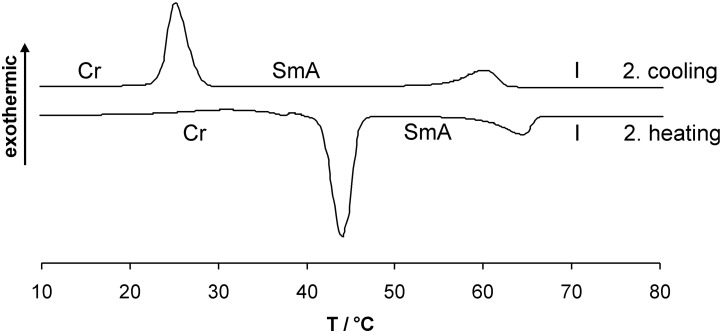
DSC curve of compound **6c** (heating/cooling rate 10 K/min).

**Figure 6:**
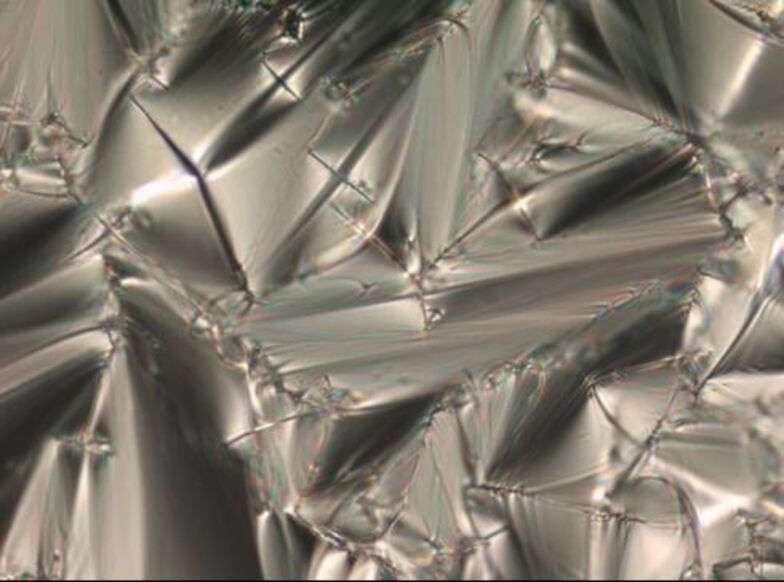
Fan-shaped texture of compound **6e** at 45 °C upon cooling from the isotropic liquid (cooling rate 10 K/min) (magnification 200×).

**Figure 7:**
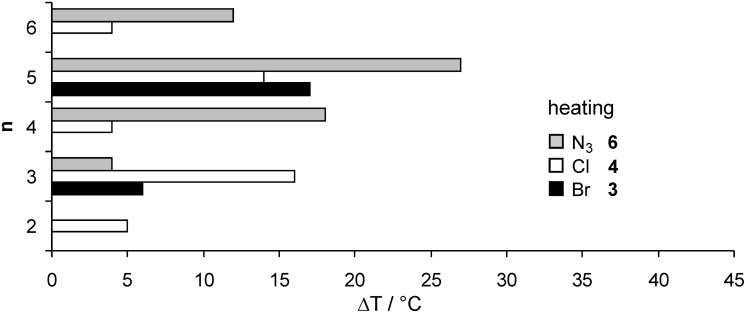
Comparison of the mesophase range Δ*T* for the different spacer lengths of compounds **3**, **4** and **6**: mesophase range upon heating (heating rate 10 K/min).

**Figure 8:**
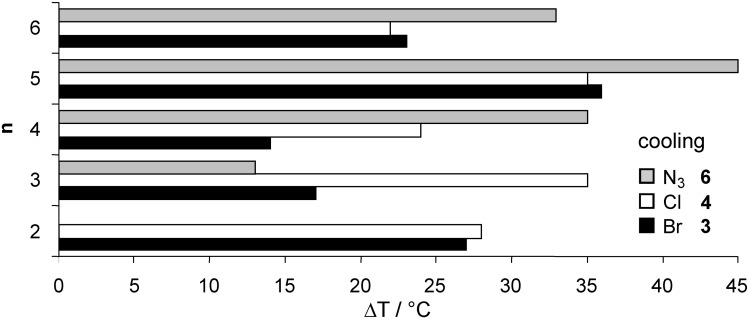
Comparison of the mesophase range Δ*T* for the different spacer lengths of compounds **3**, **4** and **6**: mesophase range upon cooling (cooling rate 10 K/min).

Whereas compound **6a** with an ethoxy spacer was non mesomorphic, enantiotropic SmA phases were detected for all other chain lengths **6b**–**e**. Compound **6d** showed an additional crystal to crystal transition. A typical DSC curve of derivative **6c** is shown in Figure 5. POM revealed fan-shaped and focal conic texture, see for example Figure 6. Figure 7 and Figure 8 reveal that due to substantial supercooling for all spacer chain lengths and terminal groups the mesophases are smaller during the heating cycle as compared to the cooling cycle. The broadest mesophase was observed for the azide derivative **6d** with Δ*T* = 27 °C upon heating and Δ*T* = 45 °C upon cooling. In comparison to the compounds with an azide as terminal group the halides (*n* = 2, 5, 6) have a lower tendency to supercooling. Whereas for azides **6** the broadest mesophase was observed for C_5_ spacer (**6d**), for chlorides **4** derivatives **4b** and **4d** with C_3_ and C_5_ spacer displayed similar mesophase width. For bromides **3** again the derivative **3d** with C_5_ spacer showed the broadest mesophase.

**Figure 9:**
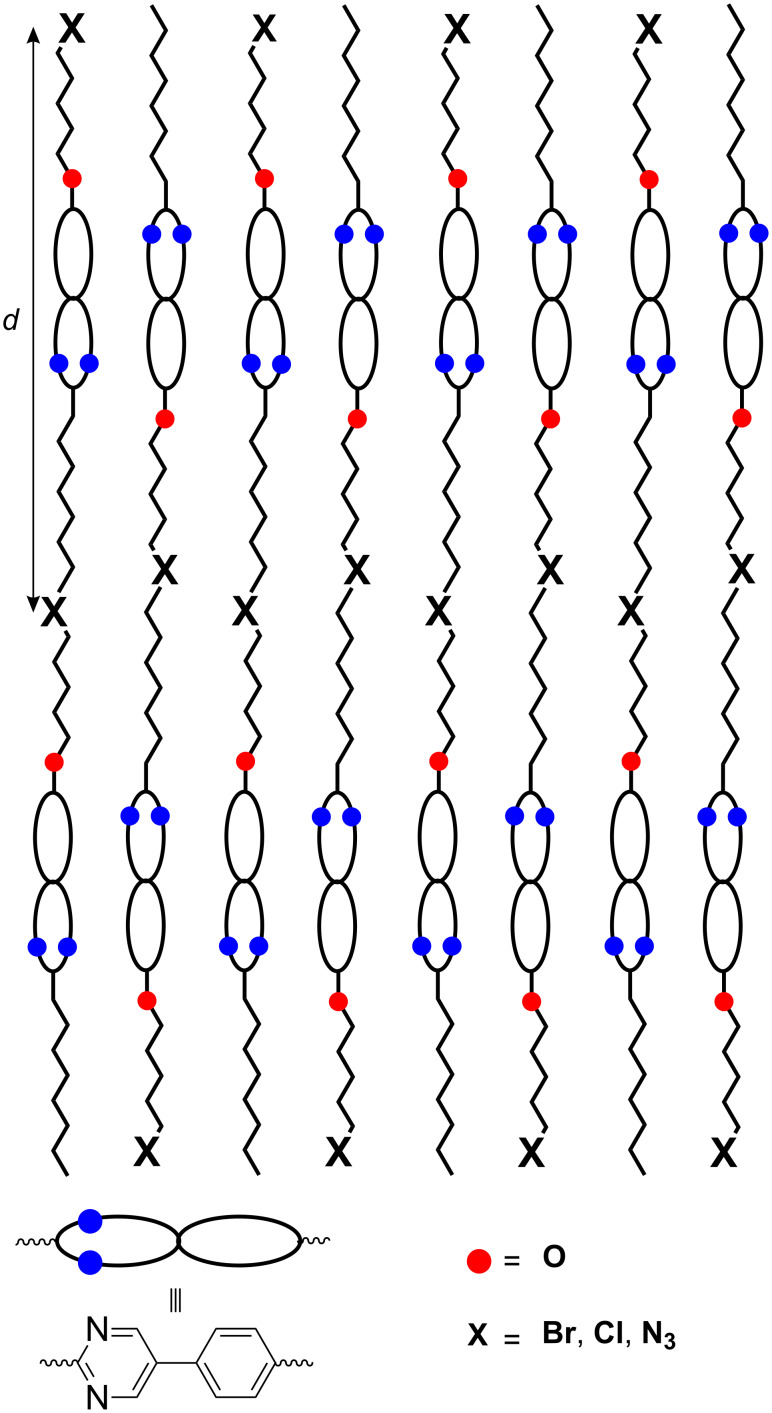
Proposed model for the layer structure.

From the X-ray data, the following model (Figure 9) of the layer structure is proposed. The *d* values obtained from the X-ray experiments fit with the molecular lengths derived from simple molecular modelling (Chem3D) [13]. For example, the XRD pattern of the azide derivative **6c** results in a layer distance of 25.5 Å, whereas the calculated length of the molecule for the most elongated conformation is 26 Å, which is clear evidence for the presence of monolayers. This leads to the assumption that the molecules might be aligned antiparallel within each smectic layer (Figure 9). Packing the molecules in this array prevents close contacts between the polar regions of the rigid core and the terminal groups. The observed maximum phase width for the C_5_ spacer regardless of the terminal group suggests that for this chain length space filling is optimal and the terminal group X can be accommodated well between the alkyl chains. This model might also explain why the mesophase is lost with strongly polar or hydrogen bonding terminal groups such as cyanides and hydroxy derivatives.

## Conclusion

It has been shown that 5-phenylpyrimidine derivatives with terminal chloro-, bromo-, azido-, hydroxy- and cyano groups separated by an alkoxy spacer chain from the aromatic core were easily synthesised by nucleophilic substitution. Depending on the terminal group and the tether lengths the formation of smectic A mesophases was observed for the chloro-, bromo- and azido derivatives. Surprisingly, the strong latent dipole moment of hydroxy and cyano derivatives and the ability of hydroxy derivatives to form hydrogen bonds seems to completely suppress the formation of mesophases. Furthermore, the polar pyrimidine ring seems to play an important role in promoting liquid crystalline properties.

## Experimental

### General

Melting points were measured on a Mettler Toledo DSC822 and are uncorrected. NMR spectra were recorded on a Bruker Avance 300 and Avance 500 spectrometer. FT-IR spectra were recorded on a Bruker Vektor22 spectrometer with MKII Golden Gate Single Reflection Diamant ATR system. Mass spectra were recorded on a Finnigan MAT 95 and a Varian MAT 711 apparatus. X-Ray powder experiments were performed on a Bruker Nanostar; software: SAXS 4.1.26. The samples were kept in Hilgenberg glass capillaries of 0.7 mm outside diameter in a temperature-controlled heating stage (±1 °C). A monochromatic Cu-K_α_1 beam (λ = 1.5405 Å) was obtained using a ceramic tube generator (1500 W) with cross-coupled Göbel-mirrors as the monochromator. The diffraction patterns were recorded on a real-time 2D-detector (HI-STAR, Bruker). The calibration of the patterns occurred with the powder pattern of Ag-Behenate. Differential scanning calorimetry (DSC) was performed using a Mettler Toledo DSC822, and polarizing optical microscopy (POM) using an Olympus BX50 polarizing microscope combined with a Linkam LTS350 hot stage and a Linkam TP93 central processor. Flash chromatography was performed using Kieselgel 60, 40–63 μm (Fluka). All solvents were dried, and reactions were performed in dried glassware. The used petroleum ether (PE) had a boiling range of 30–75 °C.

### General procedure 1

To a solution of 5-(4-hydroxyphenyl)-2-octylpyrimidine **8** (852 mg, 3.00 mmol) in 4 mL DMSO was added powdered KOH (504 mg, 9.00 mmol). After stirring for 10 min at room temperature, the α,ω-dihaloalkane (or α-bromo-ω-alkanol respectively) (3.00 mmol) was added. Stirring was continued for 4 h followed by quenching with 20 mL H_2_O and 100 mL CH_2_Cl_2_. The organic layer was dried (Na_2_SO_4_) and the solvents were evaporated. Finally the crude product was purified by flash chromatography.

### General procedure 2

A solution of bromide **3** (0.50 mmol) and NaN_3_ (81.0 mg, 1.25 mmol) in 15 mL DMF was stirred at 100 °C for 24 h. After cooling to room temperature, the reaction mixture was treated with 20 mL H_2_O and extracted with CH_2_Cl_2_ (3 × 30 mL). The combined organic layers were dried (Na_2_SO_4_), the solvent was evaporated and the crude product purified by flash chromatography.

### General procedure 3

A solution of bromide **3** (0.50 mmol) and KCN (35.8 mg, 0.55 mmol) in 4 mL EtOH/H_2_O (3:1, v/v) was stirred at 110 °C for 12 h. After cooling to room temperature, 10 mL CH_2_Cl_2_ were added and the aqueous layer was extracted with CH_2_Cl_2_ (3 × 10 mL). The combined organic layers were washed with 1 N NaOH (1 × 10 mL) and dried (Na_2_SO_4_). Finally the solvent was evaporated and the crude product purified by flash chromatography.

### 5-[4-(6-Bromohexyloxy)phenyl]-2-octylpyrimidine (**3e**)

Prepared according to general procedure (1). Experiment: 284 mg (1.00 mmol) 5-(4-hydroxyphenyl)-2-octylpyrimidine **8**, 160 μL (245 mg, 1.00 mmol) 1,6-dibromohexane, 168 mg (3.00 mmol) KOH. Flash chromatography (PE/EtOAc, 4:1, v/v; *R*_f_ = 0.86: PE/EtOAc, 1:1, v/v) gave 268 mg (0.60 mmol, 60%) of **3e** as a colourless crystalline solid. DSC: Cr 35 °C [−23.9 kJ/mol] (SmA 58 °C [−5.5 kJ/mol]) I. ^1^H NMR (300 MHz, CDCl_3_): δ = 0.88 (t, 3H *J* = 6.9 Hz, CH_3_), 1.21–1.45 (m, 10H, CH_2_), 1.49–1.58 (m, 4H, CH_2_), 1.79–1.98 (m, 6H, CH_2_), 2.96–3.01 (m, 2H, 2-CH_2_), 3.44 (t, 2H, *J* = 6.6 Hz, CH_2_Br), 4.02 (t, 2H, *J* = 6.4 Hz, OCH_2_), 6.99–7.03 (m, 2H, 3′-H, 5′-H), 7.46–7.50 (m, 2H, 2′-H, 6′-H), 8.83 (s, 2H, 4-H, 6-H) ppm. ^13^C NMR (125 MHz, CDCl_3_): δ = 14.1 (CH_3_), 22.7, 25.3, 27.9, 28.9, 29.0, 29.2, 29.4, 31.9, 32.7, 33.8 (CH_2_), 39.2 (2-CH_2_), 67.9 (OCH_2_), 115.2 (C-3′, C-5′), 128.0 (C-2′, C-6′), 126.7, 130.8 (C-1′, C-5), 154.8 (C-4, C-6), 159.9 (C-4′), 169.5 (C-2) ppm. FT-IR (ATR): 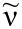 = 2916 (m), 2848 (m), 1586 (m), 1536 (m), 1515 (m), 1445 (s), 1247 (s), 1180 (m), 1116 (m), 1011 (m), 994 (m), 838 (s), 651 (m), 608 (m) cm^−1^. MS (EI, 70eV): *m/z* (%) = 446.1 (100) [M]^+^, 361.0 (20) [M−C_6_H_13_]^+^, 348.0 (78) [M + H−C_7_H_15_], 199.0 (10) 186.0 (28). C_24_H_35_BrN_2_O (447.45): calcd. C 64.42, H 7.88, N 6.26, Br 17.86; found C 64.46, H 7.88, N 6.14, Br 17.61.

### 5-[4-(6-Chlorohexyloxy)phenyl]-2-octylpyrimidine (**4e**)

Prepared according to general procedure (1). Experiment: 85.0 mg (0.30 mmol) 5-(4-hydroxyphenyl)-2-octylpyrimidine **8**, 45.0 μL (47.0 mg, 0.30 mmol) 1,6-dichlorohexane, 50.0 mg (0.90 mmol) KOH. Flash chromatography (PE/EtOAc, 5:1, v/v; *R*_f_ = 0.40: PE/EtOAc, 3:1, v/v) gave 74.0 mg (0.18 mmol, 60%) of **4e** as a colourless crystalline solid. DSC: Cr 42 °C [−25.3 kJ/mol] SmA 64 °C [−4.5 kJ/mol] I. ^1^H NMR (300 MHz, CDCl_3_): δ = 0.85–0.90 (m, 3H, CH_3_), 1.23–1.44 (m, 10H, CH_2_), 1.50–1.55 (m, 4H, CH_2_), 1.78–1.90 (m, 6H, CH_2_), 2.96–3.01 (m, 2H, 2-CH_2_), 3.56 (t, 2H, *J* = 6.7 Hz, CH_2_Cl), 4.01 (t, 2H, *J* = 6.4 Hz, OCH_2_), 6.99–7.04 (m, 2H, 3′-H, 5′-H), 7.46–7.51 (m, 2H, 2′-H, 6′-H), 8.83 (s, 2H, 4-H, 6-H) ppm. ^13^C NMR (75 MHz, CDCl_3_): δ = 14.1 (CH_3_), 22.7, 25.4, 26.6, 28.8, 29.1, 29.2, 29.4, 31.9, 32.5 (CH_2_), 39.2 (2-CH_2_), 45.0 (CH_2_Cl), 67.9 (OCH_2_), 115.3 (C-3′, C-5′), 127.9 (C-2′, C-6′), 126.7, 130.8 (C-1′, C-5), 154.5 (C-4, C-6), 159.6 (C-4′), 169.6 (C-2) ppm. FT-IR (ATR): 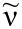 = 2917 (m), 2849 (m), 1587 (m), 1516 (m), 1446 (s), 1392 (m), 1287 (m), 1246 (s), 1181 (m), 1116 (m), 1029 (m), 994 (m), 941 (m), 838 (m), 721 (m) cm^−1^. MS (ESI): *m/z* = 403.3 [M + H]^+^, 367.3 [M–Cl]^+^. C_24_H_35_ClN_2_O (403.00): calcd. C 71.53, H 8.75, N 6.95, Cl 8.80; found C 71.38, H 8.63, N 6.81, Cl 8.93.

### 5-[4-(6-Hydroxyhexyloxy)phenyl]-2-octylpyrimidine (**5e**)

Prepared according to general procedure (1). Experiment: 568 mg (2.00 mmol) 5-(4-hydroxyphenyl)-2-octylpyrimidine **8**, 270 μL (362 mg, 2.00 mmol) 6-bromohexane-1-ol, 336 mg (6.00 mmol) KOH. Flash chromatography (PE/EtOAc, 1:1, v/v; *R*_f_ = 0.29) gave 615 mg (1.60 mmol, 80%) of **5e** as a colourless crystalline solid. Mp: 75 °C. ^1^H NMR (300 MHz, CDCl_3_): δ = 0.85–0.90 (m, 3H, CH_3_), 1.24–1.68 (m, 16H, CH_2_), 1.79–1.90 (m, 4H, CH_2_), 2.96–3.01 (m, 2H, 2-CH_2_), 3.68 (t, 2H, *J* = 6.5 Hz, CH_2_OH), 4.01 (t, 2H, *J* = 6.5 Hz, OCH_2_), 6.99–7.04 (m, 2H, 3′-H, 5′-H), 7.46–7.51 (m, 2H, 2′-H, 6′-H), 8.83 (s, 2H, 4-H, 6-H) ppm. ^13^C NMR (75 MHz, CDCl_3_): δ = 14.1 (CH_3_), 22.7, 25.6, 25.9, 28.9, 29.2, 29.5, 31.9, 32.7 (CH_2_), 39.2 (2-CH_2_), 62.8 (CH_2_OH), 68.0 (OCH_2_), 115.3 (C-3′, C-5′), 127.9 (C-2′, C-6′), 126.6, 130.8 (C-1’, C-5), 154.5 (C-4, C-6), 159.7 (C-4′), 169.6 (C-2) ppm. FT-IR (ATR): 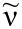 = 3303 (m, br), 2917 (s), 2848 (m), 1606 (m), 1586 (m), 1536 (m), 1516 (m), 1466 (m), 1445 (s), 1377 (m), 1291 (m), 1288 (m), 1248 (s), 1181 (m), 1118 (m), 1060 (m), 1007 (m), 994 (m), 918 (m), 838 (s), 707 (m), 652 (m) cm^−1^. MS (EI, 70eV): *m/z* (%) = 384.3 (100) [M]^+^, 299.2 (36), 286.2 (94). HRMS (ESI): *m/z* [M+H]^+^ calcd. for C_24_H_37_N_2_O_2_: 385.2850; found: 385.2856. C_24_H_36_N_2_O_2_ (384.55): calcd. C 74.96, H 9.44, N 7.28; found C 75.05, H 9.27, N 7.26.

### 5-[4-(6-Azidohexyloxy)phenyl]-2-octylpyrimidine (**6e**)

Prepared according to general procedure (2). Experiment: 224 mg (0.50 mmol)) bromide **3e**, 81.0 mg (1.25 mmol) NaN_3_. Flash chromatography (PE/EtOAc, 2:1, v/v; *R*_f_ = 0.56) gave 205 mg (0.50 mmol, quant.) of **6e** as a colourless crystalline solid. DSC: Cr_1_ 20 °C [−22.2 kJ/mol] Cr_2_ 25 °C [−0.5 kJ/mol] SmA 58 °C [−5.9 kJ/mol] I. ^1^H NMR (300 MHz, CDCl_3_): δ = 0.85–0.90 (m, 3H, CH_3_), 1.24–1.58 (m, 14H, CH_2_), 1.60–1.71 (m, 2 H, CH_2_), 1.78–1.91 (m, 4H, CH_2_), 2.97–3.01 (m, 2H, 2-CH_2_), 3.30 (t, 2H, *J* = 6.8 Hz, CH_2_N_3_), 4.02 (t, 2H, *J* = 6.4 Hz, OCH_2_), 7.00–7.04 (m, 2H, 3′-H, 5′-H), 7.47–7.51 (m, 2H, 2′-H, 6′-H), 8.83 (s, 2H, 4-H, 6-H) ppm. ^13^C NMR (75 MHz, CDCl_3_): δ = 14.1 (CH_3_), 22.7, 25.7, 26.5, 28.8, 28.9, 29.1, 29.2, 29.5, 31.9 (CH_2_), 39.2 (2-CH_2_), 51.4 (CH_2_N_3_), 67.9 (OCH_2_), 115.3 (C-3′, C-5′), 127.9 (C-2′, C-6′), 126.7, 130.8 (C-1’, C-5), 154.5 (C-4, C-6), 159.6 (C-4′), 169.6 (C-2) ppm. FT-IR (ATR): 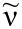 = 2921 (s), 2847 (m), 2091 (s), 1605 (m), 1587 (m), 1467 (m), 1446 (s), 1287 (m), 1246 (s), 1182 (m), 1032 (m), 993 (m), 836 (s), 654 (m) cm^−1^. MS (ESI): *m/z* = 432.3 [M + Na]^+^, 410.3 [M + H]^+^. C_24_H_35_N_5_O (409.58): calcd. C 70.38, H 8.61, N 17.10; found C 70.51, H 8.55, N 17.05.

### 5-[4-(6-Cyanohexyloxy)phenyl]-2-octylpyrimidine (**7e**)

Prepared according to general procedure (3). Experiment: 224 mg (0.50 mmol) bromide **3e**, 36.0 mg (0.55 mmol) KCN. Flash chromatography (PE/EtOAc, 4:1, v/v; *R*_f_ = 0.23) gave 142 mg (0.36 mmol, 72%) of **7e** as a colourless crystalline solid. Mp: 68 °C. ^1^H NMR (500 MHz, CDCl_3_): δ = 0.88 (t, 3H *J* = 6.9 Hz, CH_3_), 1.20–1.45 (m, 10H, CH_2_), 1.51–1.60 (m, 4H, CH_2_), 1.68–1.90 (m, 6H, CH_2_), 2.37 (t, 2H, *J* = 7.3 Hz, CH_2_CN), 2.96–3.01 (m, 2H, 2-CH_2_), 4.02 (t, 2H, *J* = 6.3 Hz, OCH_2_), 6.99–7.03 (m, 2H, 3′-H, 5′-H), 7.46–7.51 (m, 2H, 2′-H, 6′-H), 8.81 (s, 2H, 4-H, 6-H) ppm. ^13^C NMR (125 MHz, CDCl_3_): δ = 14.1 (CH_3_), 17.1, 22.7, 25.3, 25.4, 28.4, 28.9, 29.2, 29.5, 31.9 (CH_2_), 39.2 (2-CH_2_), 67.9 (OCH_2_), 115.4 (C-3′, C-5′), 119.7 (CN), 128.0 (C-2′, C-6′), 126.9, 130.8 (C-1’, C-5), 154.5 (C-4, C-6), 159.6 (C-4′), 169.7 (C-2) ppm. FT-IR (ATR): 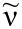 = 3035 (w), 2950 (m), 2920 (m), 2852 (m), 1608 (m) 1588 (m), 1541 (m), 1518 (m), 1440 (s), 1397 (m), 1245 (s), 1188 (s), 1048 (m), 996 (m), 836 (s), 738 (m), 706 (m), 652 (m), 556 (m) cm^−1^. MS (EI, 70eV): *m/z* (%) = 393.2 (72) [M]^+^, 350.2 (8), 308.2 (28), 395.2 (100), 185.1 (11). HRMS (ESI): *m/z* [M+H]^+^ calcd. for C_25_H_36_N_3_O: 394.2853; found: 394.2854. C_25_H_35_N_3_O (393.56): calcd. C 76.29, H 8.96, N 10.68; found C 76.15, H 8.93, N 10.51.

## Supporting Information

Supporting information includes experimental and spectroscopic data for compounds **4a**–**d**, **5d**, **6a**–**d**, **7a**–**d**, **9**, **11** and X-ray diffraction data.

File 1 Analytical data of compounds **4a**–**d**, **5d**, **6a**–**d**, **7a**–**d**, **9**, **11**.
