# Supplementary material for: Influence of spacer chain lengths and polar terminal groups on the mesomorphic properties of tethered 5-phenylpyrimidines
Source: Beilstein J Org Chem. 2009 Nov 9;5:63. doi: 10.3762/bjoc.5.63 (PMC2839790; doi:10.3762/bjoc.5.63)
Supplement: File 1 — Analytical data of compounds 4a–d, 5d, 6a–d, 7a–d, 9, 11. [file Beilstein_J_Org_Chem-05-63-s001.pdf]

Influence of spacer chain lengths and polar terminal groups on the mesomorphic properties of tethered 5-phenylpyrimidines

Gundula F. Starkulla[1], Elisabeth Kapatsina[1], Angelika Baro[1], Frank Giesselmann[2], Stefan Tussetschläger[1], Martin Kaller[1], Sabine Laschat*[1]

[1]Institut für Organische Chemie, Universität Stuttgart, Pfaffenwaldring 55, 70569 Stuttgart, Germany and [2]Institut für Physikalische Chemie, Universität Stuttgart, Pfaffenwaldring 55, 70569 Stuttgart, Germany

Email: Sabine Laschat - sabine.laschat@oc.uni-stuttgart.de

*corresponding author

Supporting Information

Analytical data of compounds **4a–d**, **5d**, **6a–d**, **7c–d**, **9**, **11**.

5-[4-(2-bromoethoxy)phenyl]-2-octylpyrimidine (3a)

844 mg (2.97 mmol) of 5-(4-hydroxyphenyl)-2-octylpyrimidine **8**, 1.23 mg (8.91 mmol) K_2CO_3 and (1.34 g, 7.12 mmol) 1,2-dibromoethane were suspended in 50 mL acetonitrile. The reaction mixture was refluxed for 12 h. After cooling to room temp., the solvent was evaporated and the solid was dissolved in 50 mL CH_2Cl_2 and 25 mL H_2O. The aqueous layer was extracted with CH_2Cl_2 (2 × 15 mL), the combined organic layers were washed with water (2 × 25 mL) and dried (Na_2SO_4). The solvent was removed under reduced pressure and the crude product was purified by flash chromatography (PE/EtOAc, 5:1, v/v; R_f = 0.34) to give 450 mg (1.15 mmol, 39%) of **3a** as a colourless crystalline solid.

DSC: Cr 29 °C [−25.8 kJ/mol] SmA 56 °C [−5.6 kJ/mol] I. ^{1}H NMR (500 MHz, $CDCl_3$): δ = 0.88 (t, 3H, J = 7.3 Hz, CH_3), 1.26–1.43 (m, 10H, CH_2), 1.81–1.89 (m, 2H, CH_2), 2.96–3.01 (m, 2H, 2-CH_2), 3.68 (t, 2H, J = 6.3 Hz, CH_2Br), 4.35 (t, 2H, J = 6.3 Hz, OCH_2), 7.02–7.07 (m, 2H, 3'-H, 5'-H), 7.48–7.53 (m, 2H, 2'-H, 6'-H), 8.82 (s, 2H, 4-H, 6-H) ppm. ^{13}C NMR (125 MHz, $CDCl_3$): δ = 14.1 (CH_3), 22.7, 28.7, 29.2, 29.5, 31.9 (CH_2), 39.2 (2-CH_2), 68.0 (OCH_2), 115.6 (C-3', C-5'), 128.1 (C-2', C-6'), 127.3, 130.8 (C-1', C-5), 154.6 (C-6, C-4), 158.9 (C-4'), 169.7 (C-2). ppm. FT-IR (ATR): ṽ = 3035 (w), 2952 (m), 2921 (m), 2848 (m), 1606 (m), 1586 (m), 1542 (m), 1517 (m), 1441 (s), 1385 (m), 1286 (m), 1241 (s), 1220 (m), 1185 (m), 1075 (m), 1036 (m), 996 (m), 935 (s), 830 (s), 806 (m), 782 (w), 704 (m), 654 (m), 608 (w), 575 (w) cm^{-1}. MS (ESI): m/z = 415.1 $[M+Na]^+$, 391.1 $[M+H]^+$. HRMS (ESI): *m/z* $[M^+]$ calc. for $C_{21}H_{28}{}^{79}BrN_2O$: 391.1380; found: 391.1391. $C_{20}H_{27}BrN_2O$ (391.35): calc. C 61.38, H 6.95, N 7.16, Br 20.42; found C 61.31, H 6.97, N 7.09, Br 20.52.

5-[4-(3-bromopropoxy)phenyl]-2-octylpyrimidine (3b)

Prepared according to general procedure (1). Experiment: 853 mg (3.00 mmol) 5-(4-hydroxyphenyl)-2-octylpyrimidine **8**, 310 µL (604 mg, 3.00 mmol) 1,3-dibromopropane, 505 mg (9.00 mmol) KOH. Flash chromatography (PE/EtOAc, 4:1, v/v; R_f = 0.43) gave 285 mg (0.70 mmol, 23%) of **3b** as a colourless crystalline solid. DSC: Cr 35 °C [−13.9 kJ/mol] SmA 52 °C [−3.2 kJ/mol] I. ^{1}H NMR (300 MHz, $CDCl_3$): δ = 0.85–0.90 (m, 3H, CH_3), 1.24–1.46 (m, 10H, CH_2), 1.80–1.90 (m, 2H, CH_2), 2.31–2.40 (m, 2H, CH_2), 2.97–3.02 (m, 2H, 2-CH_2), 3.63 (t, 2H, J = 6.4 Hz, CH_2Br), 4.17 (t, 2H, J = 5.8 Hz, OCH_2), 7.01–7.06 (m, 2H, 3'-H, 5'-H), 7.48–7.52 (m, 2H, 2'-H, 6'-H), 8.83 (s, 2H, 4-H, 6-H) ppm. ^{13}C NMR (75 MHz, $CDCl_3$): δ = 14.1 (CH_3), 22.7, 28.9, 29.2, 29.5, 29.9, 31.9, 32.2 (CH_2), 39.2 (2-CH_2), 65.4 (OCH_2), 115.4 (C-3', C-5'), 128.0 (C-2', C-6'), 127.2, 130.7 (C-1', C-5), 154.6 (C-4, C-6), 159.2 (C-4'), 169.7 (C-2) ppm. FT-IR (ATR): ṽ = 2921 (s), 2851 (m), 1607 (m), 1589 (m), 1541 (m), 1517 (m), 1443 (s), 1387 (m), 1284 (m), 1241 (s), 1184 (s), 1089 (m), 1033

(s), 998 (m), 932 (s), 830 (s), 811 (m), 768 (m), 707 (m), 654 (m), 618 (m), 551 (m) cm^{-1}. MS (EI, 70eV): *m/z* (%) = 406.1 (76) [M (^{81}Br)]$^+$, 361.1 (12) [M − C_3H_{10}]$^+$, 321.0 (28) [M − C_6H_{13}]$^+$, 308.0 (100) [M + H − C_7H_{15}]$^+$,185.1 (32). HRMS (ESI): *m/z* [M$^+$] calc. for $C_{21}H_{30}{}^{79}BrN_2O$: 405.1536; found: 405.1535; calc. for $C_{21}H_{30}{}^{81}BrN_2O$: 407.1518; found: 407.1516.

5-[4-(4-bromobutoxy)phenyl]-2-octylpyrimidine (3c)

Prepared according to general procedure (1). Experiment: 853 mg (3.00 mmol) 5-(4-hydroxyphenyl)-2-octylpyrimidine **8**, 360 µL (648 mg, 3.00 mmol) 1,4-dibromobutane, 505 mg (9.00 mmol) KOH. Flash chromatography (PE/EtOAc, 5:1, v/v; R_f = 0.47) gave 670 mg (1.59 mmol, 53%) of **3c** as a colourless crystalline solid. DSC: Cr 49 °C [−24.1 kJ/mol] (SmA 63 °C [−5.0 kJ/mol]) I. ^{1}H NMR (300 MHz, $CDCl_3$): δ = 0.85–0.90 (m, 3H, CH_3), 1.23–1.46 (m, 10H, CH_2), 1.80–1.90 (m, 2H, CH_2), 1.94–2.14 (m, 4H, CH_2), 2.96–3.02 (m, 2H, 2-CH_2), 3.51 (t, 2H, *J* = 6.5 Hz, CH_2Br), 4.05 (t, 2H, *J* = 5.9 Hz, OCH_2), 6.99–7.04 (m, 2H, 3’-H, 5’-H), 7.47–7.52 (m, 2H, 2’-H, 6’-H), 8.83 (m, 2H, 4-H, 6-H) ppm. ^{13}C NMR (75 MHz, $CDCl_3$): δ = 14.1 (CH_3), 22.7, 27.8, 28.8, 29.2, 29.42, 29.44, 31.9, 33.3 (CH_2), 39.2 (2-CH_2), 67.0 (OCH_2), 115.3 (C-3’, C-5’), 128.0 (C-2’, C-6’), 127.0, 130.8 (C-1’, C-5), 154.5 (C-4, C-6), 159.4 (C-4’), 169.7 (C-2) ppm. FT-IR (ATR): $\tilde{\nu}$ = 2949 (m), 2919 (s), 2852 (m), 1606 (m), 1540 (m), 1517 (m), 1439 (s), 1398 (m), 1275 (m), 1245 (s), 1187 (s), 1047 (m), 996 (m), 836 (s), 816 (m), 739 (m), 652 (s), 608 (m), 557 (s), 528 (m) cm^{-1}. MS (EI, 70eV): *m/z* (%) = 420.1 (92) [M (^{81}Br)]$^+$, 335.0 (28) [M − C_6H_{13}]$^+$, 320.0 (100) [M + H − C_7H_{15}]$^+$, 199.1 (12), 185.1 (28), 55.1 (16). $C_{22}H_{31}BrN_2O$ (419.40): calc. C 63.00, H 7.45, N 6.68, Br 19.05; found C 63.18, H 7.39, N 6.40, Br 18.90.

5-[4-(5-bromopentyloxy)phenyl]-2-octylpyrimidine (3d)

Prepared according to general procedure (1). Experiment: 561 mg (2.00 mmol) 5-(4-hydroxyphenyl)-2-octylpyrimidine **8**, 300 µL (460 mg, 2.00 mmol) 1,5-dibromopentane,

336 mg (6.00 mmol) KOH. Flash chromatography (PE/EtOAc, 6:1, v/v; $R_f = 0.43$) gave 453 mg (1.05 mmol, 53%) of **3d** as a colourless crystalline solid. DSC: Cr 24 °C [−16.7 kJ/mol] SmA 60 °C [−5.3 kJ/mol] I. ^{1}H NMR (300 MHz, $CDCl_3$): δ = 0.85–0.90 (m, 3H, CH_3), 1.23–1.47 (m, 10H, CH_2), 1.60–1.90 (m, 8H, CH_2), 2.96–3.02 (m, 2H, 2-CH_2), 3.46 (t, 2H, J = 6.7 Hz, CH_2Br), 4.03 (t, 2H, J = 6.3 Hz, OCH_2), 6.99–7.04 (m, 2H, 3'-H, 5'-H), 7.46–7.51 (m, 2H, 2'-H, 6'-H), 8.82 (s, 2H, 4-H, 6-H) ppm. ^{13}C NMR (75 MHz, $CDCl_3$): δ = 14.1 (CH_3), 22.7, 24.8, 26.9, 28.4, 28.8, 29.2, 29.4, 31.9, 32.4, 33.5 (CH_2), 39.2 (2-CH_2), 67.7 (OCH_2), 115.4 (C-3', C-5'), 127.9 (C-2', C-6'), 126.8, 130.8 (C-1', C-5), 154.5 (C-4, C-6), 159.5 (C-4'), 169.6 (C-2) ppm. FT-IR (ATR): ṽ = 2921 (s), 2852 (m), 1607 (m), 1541 (m), 1516 (m), 1442 (s), 1394 (m), 1284 (m), 1245 (s), 1185 (s), 1041 (m), 1014 (m), 995 (m), 933 (m), 834 (s), 810 (s), 730 (m), 652 (s), 609 (m), 556 (m) cm^{-1}. MS (EI, 70eV): m/z (%) = 434.2 (100) [M (^{81}Br)]$^+$, 349.0 (20) [M − C_6H_{13}]$^+$, 336.0 (88) [M + H − C_7H_{15}]$^+$, 186.0 (28). $C_{23}H_{33}BrN_2O$ (433.42): calc. C 63.74, H 7.67, N 6.46, Br 18.44; found C 63.97, H 7.64, N 6.44, Br 18.14.

5-[4-(allyloxy)phenyl]-2-octylpyrimidine (9)

Byproduct of **3b**. Flash chromatography (PE/EtOAc, 4:1, v/v; $R_f = 0.52$) gave 266 mg (0.82 mmol, 27%) of **9** as a colourless crystalline solid. DSC: Cr 37 °C [−23.0 kJ/mol] SmA 70 °C [−4.9 kJ/mol] I. ^{1}H NMR (300 MHz, $CDCl_3$): δ = 0.85–0.90 (m, 3H, CH_3), 1.23–1.46 (m, 10H, CH_2), 1.80–1.90 (m, 2H, CH_2), 2.96–3.02 (m, 2H, 2-CH_2), 4.58–4.61 (m, 2H, OCH_2), 5.30–5.35 (m, 1H, CH=CH_2), 5.41–5.48 (m, 1H, CH=CH_2), 6.01–6.14 (m, 1H, CH=CH_2), 7.02–7.07 (m, 2H, 3'-H, 5'-H), 7.47–7.52 (m, 2H, 2'-H, 6'-H), 8.83 (m, 2H, 4-H, 6-H) ppm. ^{13}C NMR (75 MHz, $CDCl_3$): δ = 14.1 (CH_3), 22.7, 28.9, 29.2, 29.4, 31.9 (CH_2), 32.2, 39.2 (2-CH_2), 68.9 (OCH_2), 115.6 (C-3', C-5'), 118.0 (CH=CH$_2$), 127.9 (C-2', C-6'), 127.0, 130.8 (C-1', C-5), 132.9 (CH=CH_2), 154.5 (C-4, C-6), 159.1 (C-4'), 169.7 (C-2) ppm. FT-IR (ATR): ṽ = 2954 (m), 2916 (s), 2848 (m), 1607 (m), 1587 (m), 1537 (m), 1516 (m),

1445 (s), 1426 (s), 1362 (m), 1246 (s), 1182 (s), 1118 (m), 1011 (m), 993 (s), 935 (s), 839 (s), 827 (s), 719 (m), 652 (m), 559 (m) cm^{-1}. MS (EI, 70eV): *m/z* (%) = 324.2 (100) $[M]^+$, 239.1 (38) $[M - C_6H_{13}]^+$, 226.1 (100) $[M + H - C_7H_{15}]^+$, 185.1 (61). $C_{21}H_{28}N_2O$ (324.46): calc. C 77.74, H 8.70, N 8.63; found C 77.59, H 8.67, N 8.57.

4-(allyloxy)-4'-octylbiphenyl (11)

To a solution of 297 mg (1.05 mmol) 4-(hydroxyl)-4'-octylbiphenyl **10** in 2 mL DMSO was added powdered KOH (177 mg, 3.15 mmol). After 10 min of stirring at room temp., allybromide 90.0 µL (127 mg, 1.05 mmol) was added and the reaction mixture was stirred for further 4 h at room temp., followed by quenching with 10 mL H_2O and 50 mL CH_2Cl_2. The organic layer was dried (Na_2SO_4) and the solvents were evaporated. The crude product was purified by flash chromatography (PE/EtOAc, 30:1, v/v) to give 163 mg (0.51 mmol, 49%) of the allylether **11** as a colourless crystalline solid.

R_f = 0.52 (PE/EtOAc, 30:2, v/v). Mp: 93 °C. ^1H NMR (300 MHz, $CDCl_3$): δ = 0.85–0.90 (m, 3H, CH_3), 1.23–1.40 (m, 10H, CH_2), 1.58–1.68 (m, 2H, CH_2), 2.60–2.65 (m, 2H, 4'-CH_2), 4.56–4.59 (m, 2H, OCH_2), 5.29–5.30 (m, 1H, CH=CH_2), 5.40–5.47 (m, 1H, CH=CH_2), 6.02–6.13 (m, 1H, C*H*=CH_2), 6.96–6.99 (m, 2H, 3-H, 5-H), 7.21–7.23 (m, 2H, 3'-H, 5'-H), 7.43–7.53 (m, 4H, 2-H, 6-H, 2'-H, 6'-H) ppm. ^{13}C NMR (75 MHz, $CDCl_3$): δ = 14.1 (CH_3), 22.7, 29.3, 29.4, 29.5, 31.5, 31.9 (CH_2), 35.6 (4'-CH_2), 68.9 (OCH_2), 115.0 (C-3, C-5), 117.7 (CH=*C*H_2), 126.5, 127.9 (C-2, C-6, C-2', C-6'), 128.8 (C-3', C-5'), 133.3 (*C*H=CH_2), 133.9, 138.1 (C-1, C-1'), 141.5 (C-4'), 157.9 (C-4) ppm. FT-IR (ATR): ṽ = 2956 (m), 2917 (s), 2848 (m), 1606 (m), 1497 (m), 1456 (m), 1410 (m), 1382 (m), 1272 (m), 1252 (m), 1205 (m), 1182 (m), 1118 (m), 1026 (m), 996 (m), 920 (m), 808 (s), 784 (m), 721 (m) cm^{-1}. MS (EI, 70eV): *m/z* (%) = 322.2 (100) $[M]^+$, 281.2 (84) $[M - CH_2CH{=}CH_2]$. $C_{23}H_{30}O$ (322.48): calc. C 85.66, H 9.38 found C 85.82, H 8.26.

5-[4-(2-chloroethoxy)phenyl]-2-octylpyrimidine (4a)

Prepared according to general procedure (1). Experiment: 114 mg (0.40 mmol) 5-(4-hydroxyphenyl)-2-octylpyrimidine **8**, 32.0 µL (40.0 mg, 0.40 mmol) 1,2-dichloroethane, 67.0 mg (1.20 mmol) KOH. Flash chromatography (PE/EtOAc, 3:1, v/v; $R_f = 0.38$) gave 43.0 mg (0.12 mmol, 31%) of **4a** as a colourless crystalline solid. DSC: Cr 25 °C [−18.7 kJ/mol] SmA 53 °C [−1.5 kJ/mol] I. ^{1}H NMR (300 MHz, $CDCl_3$): δ = 0.85–0.90 (m, 3H, CH_3), 1.23–1.46 (m, 10H, CH_2), 1.80–1.90 (m, 2H, CH_2), 2.97–3.02 (m, 2H, 2-CH_2), 3.85 (t, 2H, J = 5.9 Hz, CH_2Cl), 4.29 (t, 2H, J = 5.9 Hz, OCH_2), 7.03–7.07 (m, 2H, 3’-H, 5’-H), 7.48–7.53 (m, 2H, 2’-H, 6’-H), 8.83 (s, 2H, 4-H, 6-H) ppm. ^{13}C NMR (125 MHz, $CDCl_3$): δ = 14.1 (CH_3), 22.7, 28.8, 29.2, 29.4, 31.9 (CH_2), 39.1 (2-CH_2), 41.8 (CH_2Cl), 68.2 (OCH_2), 115.6 (C-3’, C-5’), 128.1 (C-2’, C-6’), 127.6, 130.8 (C-1’, C-5), 154.5 (C-4, C-6), 158.8 (C-4’), 169.7 (C-2) ppm. FT-IR (ATR): ṽ = 2953 (m), 2914 (m), 2848 (m), 1608 (m), 1587 (m), 1536 (m), 1517 (m), 1445 (s), 1380 (m), 1337 (m), 1289 (m), 1248 (s), 1181 (m), 1116 (m), 1032 (s), 996 (m), 941 (m), 830 (s), 795 (m), 754 (m), 721 (m) cm^{-1}. MS (ESI): m/z = 369.2 $[M + Na]^+$, 347.2 $[M + H]^+$. $C_{20}H_{27}ClN_2O$ (346.89): calc. C 69.25, H 7.85, N 8.08, Cl 10.22; found C 69.15, H 7.79, N 7.81, Cl 10.22.

5-[4-(3-chloropropoxy)phenyl]-2-octylpyrimidine (4b)

Prepared according to general procedure (1). Experiment: 284 mg (1.00 mmol) 5-(4-hydroxyphenyl)-2-octylpyrimidine **8**, 90.0 µL (113 mg, 1.00 mmol) 1,3-dichloropropane, 112 mg (2.00 mmol) KOH. Flash chromatography (PE/EtOAc, 4:1, v/v; $R_f = 0.38$) gave 175 mg (0.49 mmol, 49%) of **4b** as a colourless crystalline solid. DSC: Cr 23 °C [−11.8 kJ/mol] SmA 58 °C [−4.2 kJ/mol] I. ^{1}H NMR (500 MHz, $CDCl_3$): δ = 0.87–0.90 (m, 3H, CH_3), 1.23–1.44 (m, 10H, CH_2), 1.83–1.88 (m, 2H, CH_2), 2.25–2.30 (m, 2H, OCH_2CH_2) 2.98–3.01 (m, 2H, 2-CH_2), 3.77 (t, 2H, J = 6.3 Hz, CH_2Cl), 4.29 (t, 2H, J = 5.7 Hz, OCH_2), 7.02–7.05 (m, 2H, 3’-H, 5’-H), 7.48–7.51 (m, 2H, 2’-H, 6’-H), 8.83 (s, 2H, 4-H, 6-H) ppm.

^{13}C NMR (125 MHz, $CDCl_3$): δ = 14.1 (CH_3), 22.7, 28.8, 29.2, 29.4, 29.5, 31.9, 32.2 (CH_2), 39.1 (2-CH_2), 41.4 (CH_2Cl), 64.5 (OCH_2), 115.5 (C-3', C-5'), 128.0 (C-2', C-6'), 127.1, 130.8 (C-1', C-5), 154.5 (C-4, C-6), 159.3 (C-4'), 169.7 (C-2) ppm. FT-IR (ATR): $\tilde{\nu}$ = 2955 (m), 2922 (m), 2848 (m), 1606 (m), 1587 (m), 1538 (m), 1515 (m), 1444 (s), 1394 (m), 1286 (m), 1289 (m), 1244 (s), 1182 (m), 1117 (m), 1036 (m), 997 (m), 949 (m), 834 (s), 709 (m) cm^{-1}. MS (ESI): m/z = 361.2 $[M + H]^+$, 325.2 $[M + H - Cl]^+$. $C_{21}H_{29}ClN_2O$ (360.92): calc. C 69.88, H 8.10, N 7.76, Cl 9.82; found C 69.88, H 8.07, N 7.60, Cl 9.86.

5-[4-(4-chlorobutoxy)phenyl]-2-octylpyrimidine (4c)

Prepared according to general procedure (1). Experiment: 114 mg (0.40 mmol) 5-(4-hydroxyphenyl)-2-octylpyrimidine **8**, 50.0 µL (51.0 mg, 0.40 mmol) 1,4-dichlorobutane, 67.0 mg (1.20 mmol) KOH. Flash chromatography (PE/EtOAc, 5:1, v/v; R_f = 0.38: PE/EtOAc, 1:1, v/v) gave 105 mg (0.28 mmol, 70%) of **4c** as a colourless crystalline solid. DSC: Cr 46 °C [−23.5 kJ/mol] SmA 70 °C [−4.9 kJ/mol] I. ^1H NMR (300 MHz, $CDCl_3$): δ = 0.85–0.90 (m, 3H, CH_3), 1.23–1.46 (m, 10H, CH_2), 1.80–1.90 (m, 2H, CH_2), 1.96–2.02 (m, 4H, CH_2), 2.96–3.01 (m, 2H, 2-CH_2), 3.64 (t, 2H, J = 6.2 Hz, CH_2Cl), 4.06 (t, 2H, J = 5.7 Hz, OCH_2), 6.99–7.04 (m, 2H, 3'-H, 5'-H), 7.47–7.52 (m, 2H, 2'-H, 6'-H), 8.83 (s, 2H, 4-H, 6-H) ppm. ^{13}C NMR (63 MHz, $CDCl_3$): δ = 14.1 (CH_3), 22.7, 26.6, 28.9, 29.2, 29.3, 29.5, 31.9 (CH_2), 39.2 (2-CH_2), 44.7 (CH_2Cl), 67.2 (OCH_2), 115.3 (C-3', C-5'), 128.0 (C-2', C-6'), 126.9, 130.8 (C-1', C-5), 154.5 (C-4, C-6), 159.4 (C-4'), 169.6 (C-2) ppm. FT-IR (ATR): $\tilde{\nu}$ = 2917 (s), 2850 (s), 1738 (m), 1585 (m), 1499 (m), 1432 (m), 1337 (m), 1311 (m), 1262 (m), 1248 (m), 1193 (s), 1155 (m), 1100 (s), 1062 (m), 1017 (m), 995 (m), 941 (m), 800 (m), 755 (m), 716 (m) cm^{-1}. MS (ESI): m/z = 397.2 $[M + Na]^+$, 375.2 $[M + H]^+$. $C_{22}H_{31}ClN_2O$ (374.95): calc. C 70.47, H 8.33, N 7.47, Cl 9.46; found C 70.42, H 8.33, N 7.30, Cl 9.74.

5-[4-(5-chloropentyloxy)phenyl]-2-octylpyrimidine (4d)

Prepared according to general procedure (1). Experiment: 171 mg (0.60 mmol) 5-(4-hydroxyphenyl)-2-octylpyrimidine **8**, 80.0 µL (85.0 mg, 0.40 mmol) 1,5-dichloropentane, 110 mg (1.80 mmol) KOH. Flash chromatography (PE/EtOAc, 3:1, v/v; $R_f = 0.68$) gave 72.0 mg (0.19 mmol, 31%) of **4d** as a colourless crystalline solid. DSC: Cr 27 °C [−17.0 kJ/mol] SmA 62 °C [−4.8 kJ/mol] I. ^{1}H NMR (300 MHz, $CDCl_3$): δ = 0.85–0.89 (m, 3H, CH_3), 1.23–1.44 (m, 10H, CH_2), 1.50–1.71 (m, 2H, CH_2), 1.81–1.92 (m, 6H, CH_2), 2.96–3.01 (m, 2H, 2-CH_2), 3.58 (t, 2H, J = 6.6 Hz, CH_2Cl), 4.03 (t, 2H, J = 6.4 Hz, OCH_2), 6.99–7.04 (m, 2H, 3’-H, 5’-H), 7.47–7.51 (m, 2H, 2’-H, 6’-H), 8.83 (s, 2H, 4-H, 6-H) ppm. ^{13}C NMR (125 MHz, $CDCl_3$): δ = 14.1 (CH_3), 22.7, 23.6, 28.5, 28.8, 29.2, 29.5, 31.9, 32.3 (CH_2), 39.2 (2-CH_2), 44.8 (CH_2Cl), 67.8 (OCH_2), 115.4 (C-3’, C-5’), 128.0 (C-2’, C-6’), 126.8, 130.8 (C-1’, C-5), 154.5 (C-4, C-6), 159.6 (C-4’), 169.6 (C-2) ppm. FT-IR (ATR): ṽ = 2920 (m), 2853 (m), 1607 (m), 1516 (m), 1587 (m), 1540 (m), 1517 (m), 1441 (s), 1396 (m), 1275 (m), 1244 (s), 1186 (s), 1044 (m), 996 (m), 934 (m), 834 (s), 731 (m), 653 cm^{-1}. MS (ESI): m/z = 411.2 $[M + Na]^+$, 389.2 $[M + H]^+$. $C_{23}H_{33}ClN_2O$ (388.97): calc. C 71.02, H 8.55, N 7.20, Cl 9.11; found C 70.76, H 8.52, N 7.03, Cl 9.38.

5-[4-(5-hydroxypentyloxy)phenyl]-2-octylpyrimidine (5d)

Prepared according to general procedure (1). Experiment: 142 mg (0.50 mmol) 5-(4-hydroxyphenyl)-2-octylpyrimidine **8**, 60.0 µL (84.0 mg, 0.50 mmol) 5-bromopentane-1-ol, 84.0 mg (1.50 mmol) KOH. Flash chromatography (PE/EtOAc, 1:2, v/v; $R_f = 0.47$) gave 139 mg (0.38 mmol, 76%) of **5d** as a colourless crystalline solid. Mp: 76 °C. ^{1}H NMR (300 MHz, $CDCl_3$): δ = 0.85–0.90 (m, 3H, CH_3), 1.24–1.46 (m, 10H, CH_2), 1.50–1.70 (m, 4H, CH_2), 1.81–1.90 (m, 4H, CH_2), 2.98–3.08 (m, 2H, 2-CH_2), 3.70 (t, 2H, J = 6.3 Hz, CH_2OH), 4.03 (t, 2H, J = 6.4 Hz, OCH_2), 6.99–7.04 (m, 2H, 3’-H, 5’-H), 7.47–7.51 (m, 2H, 2’-H, 6’-H), 8.85 (s, 2H, 4-H, 6-H) ppm. ^{13}C NMR (75 MHz, $CDCl_3$): δ = 14.1 (CH_3), 22.4, 22.7, 28.9,

29.0, 29.2, 29.4, 31.9, 32.4 (CH_2), 39.2 (2-CH_2), 62.7 (CH_2OH), 68.1 (OCH_2), 115.4 (C-3', C-5'), 127.9 (C-2', C-6'), 126.7, 130.8 (C-1', C-5), 154.5 (C-4, C-6), 159.6 (C-4'), 169.6 (C-2) ppm. FT-IR (ATR): ṽ = 3271 (m, br), 2915 (m), 2848 (m), 1607 (m), 1588 (m), 1536 (m), 1518 (m), 1469 (m), 1446 (s), 1390 (m), 1291 (m), 1244 (s), 1184 (m), 1121 (m), 1099 (m), 1058 (s), 1029 (s), 1007 (m), 976 (m), 904 (m), 831 (m), 704 (m), 653 (m) cm^{-1}. MS (ESI): *m/z* = 393.3 $[M + Na]^+$, 371.3 $[M + H]^+$. $C_{23}H_{34}N_2O_2$ (370.53): calc. C 74.55, H 9.25, N 7.56; found C 74.28, H 9.16, N 7.26.

5-[4-(2-azidoethoxy)phenyl]-2-octylpyrimidine (6a)

Prepared according to general procedure (2). Experiment: 117 mg (0.30 mmol) bromide **3a**, 50.0 mg (0.75 mmol) NaN_3. Flash chromatography (PE/EtOAc, 3:1, v/v; $R_f = 0.29$) gave 78.0 mg (0.22 mmol, 74%) of **6a** as a colourless crystalline solid. Mp: 44 °C. ^1H NMR (300 MHz, $CDCl_3$): $\delta = 0.86–0.90$ (m, 3H, CH_3), 1.23–1.45 (m, 10H, CH_2), 1.80–1.90 (m, 2H, CH_2), 2.97–3.02 (m, 2H, 2-CH_2), 3.64 (t, 2H, $J = 5.0$ Hz, CH_2N_3), 4.21 (t, 2H, $J = 5.0$ Hz, OCH_2), 7.03–7.08 (m, 2H, 3'-H, 5'-H), 7.49–7.54 (m, 2H, 2'-H, 6'-H), 8.83 (s, 2H, 4-H, 6-H) ppm. ^{13}C NMR (75 MHz, $CDCl_3$): $\delta = 14.1$ (CH_3), 22.7, 28.8, 29.2, 29.4, 31.9 (CH_2), 39.1 (2-CH_2), 50.1 (CH_2N_3), 67.2 (OCH_2), 115.5 (C-3', C-5'), 128.1 (C-2', C-6'), 127.5, 130.8 (C-1', C-5), 154.6 (C-4, C-6), 158.9 (C-4'), 169.6 (C-2) ppm. FT-IR (ATR): ṽ = 2915 (m), 2848 (m), 2111 (s), 1606 (m), 1587 (m), 1538 (m), 1516 (m), 1445 (s), 1284 (m), 1379 (m), 1356 (m), 1289 (s), 1249 (s), 1183 (s), 1118 (m), 1052 (m), 997 (m), 839 (s), 805 (m), 721 (m), 707 (m), 651 (m), 615 (m) cm^{-1}. MS (ESI): *m/z* = 354.2 $[M + H]^+$, 326.2 $[M + H - N_2]^+$. $C_{20}H_{27}N_5O$ (353.46): calc. C 67.96, H 7.70, N 19.81; found C 68.11, H 7.69, N 19.56.

5-[4-(3-azidopropoxy)phenyl]-2-octylpyrimidine (6b)

Prepared according to general procedure (2). Experiment: 81.0 mg (0.20 mmol) bromide **3b**, 33.0 mg (0.50 mmol) NaN_3. Purified by flash chromatography (PE/EtOAc, 5:1, v/v; $R_f = 0.29$) gave 73.0 mg (0.20 mmol, quant.) of **6b** as a colourless crystalline solid. DSC: Cr 34 °C

[−13.7 kJ/mol] SmA 47 °C [−2.9 kJ/mol] I. ^{1}H NMR (300 MHz, $CDCl_3$): δ = 0.85–0.90 (m, 3H, CH_3), 1.23–1.46 (m, 10H, CH_2), 1.80–1.90 (m, 2H, CH_2), 2.05–2.13 (m, 2H, CH_2), 2.97–3.01 (m, 2H, 2-CH_2), 3.55 (t, 2H, J = 6.6 Hz, CH_2N_3), 4.11 (t, 2H, J = 6.0 Hz, OCH_2), 7.00–7.05 (m, 2H, 3'-H, 5'-H), 7.47–7.52 (m, 2H, 2'-H, 6'-H), 8.83 (s, 2H, 4-H, 6-H) ppm. ^{13}C NMR (75 MHz, $CDCl_3$): δ = 14.1 (CH_3), 22.7, 28.7, 28.8, 29.1, 29.2, 29.4, 31.8 (CH_2), 39.2 (2-CH_2), 48.2 (CH_2N_3), 64.6 (OCH_2), 115.3 (C-3', C-5'), 128.0 (C-2', C-6'), 127.2, 130.7 (C-1', C-5), 154.5 (C-4, C-6), 159.2 (C-4'), 169.7 (C-2) ppm. FT-IR (ATR): ṽ = 2922 (s), 2852 (m), 2096 (s), 1608 (m), 1541 (m), 1517 (m), 1442 (s), 1284 (m), 1242 (s), 1185 (s), 1041 (m), 965 (m), 833 (s), 811 (m), 654 (m), 635 (m), 612 (m) cm^{-1}. MS (ESI): m/z = 390.2 $[M + Na]^+$, 368.3 $[M + H]^+$, 340.2 $[M + H - N_2]^+$. HRMS (ESI): m/z $[M + H]^+$ ber. für $C_{21}H_{30}N_5O$: 368.2445; found: 368.2452.

5-[4-(4-azidobutoxy)phenyl]-2-octylpyrimidine (6c)

Prepared according to general procedure (2). Experiment: 100 mg (0.24 mmol) bromide **3c**, 39.0 mg (0.60 mmol) NaN_3. Flash chromatography (PE/EtOAc, 5:1, v/v; R_f = 0.40) gave 92.0 mg (0.24 mmol, quant.) of **6c** as a colourless crystalline solid. DSC: Cr 28 °C [−17.8 kJ/mol] SmA 63 °C [−5.3 kJ/mol] I. ^{1}H NMR (300 MHz, $CDCl_3$): δ = 0.85–0.90 (m, 3H, CH_3), 1.23–1.47 (m, 10H, CH_2), 1.77–1.97 (m, 6H, CH_2), 2.97–3.02 (m, 2H, 2-CH_2), 3.39 (t, 2H, J = 6.6 Hz, CH_2N_3), 4.05 (t, 2H, J = 5.9 Hz, OCH_2), 6.99–7.04 (m, 2H, 3'-H, 5'-H), 7.47–7.52 (m, 2H, 2'-H, 6'-H), 8.83 (s, 2H, 4-H, 6-H) ppm. ^{13}C NMR (75 MHz, $CDCl_3$): δ = 14.1 (CH_3), 22.7, 25.7, 26.5, 28.9, 29.2, 29.5, 31.9 (CH_2), 39.1 (2-CH_2), 51.2 (CH_2N_3), 67.3 (OCH_2), 115.1 (C-3', C-5'), 128.0 (C-2', C-6'), 126.9, 130.8 (C-1', C-5), 154.5 (C-4, C-6), 159.4 (C-4'), 169.6 (C-2) ppm. FT-IR (ATR): ṽ = 2916 (m), 2848 (m), 2098 (s), 1607 (m), 1587 (m), 1537 (m), 1515 (m), 1469 (m), 1445 (s), 1393 (m), 1352 (m), 1285 (s), 1245 (s), 1182 (s), 1116 (m), 1055 (m), 950 (m), 836 (s), 653 (m) cm^{-1}. MS (ESI): m/z = 382.3 $[M + H]^+$, 352.2 $[M - C_2H_5]^+$. $C_{22}H_{31}N_5O$ (381.51): calc. C 69.26, H 8.19, N 18.26; found C 69.11, H 8.29, N 18.20.

5-[4-(5-azidopentyloxy)phenyl]-2-octylpyrimidine (6d)

Prepared according to general procedure (2). Experiment: 174 mg (0.40 mmol) bromide **3d** 66.0 mg (1.00 mmol) NaN_3. Flash chromatography (PE/EtOAc, 5:1, v/v; $R_f = 0.29$) gave 158 mg (0.40 mmol, quant.) of **6d** as a colourless crystalline solid. DSC: Cr_1 1 °C [−0.6 kJ/mol] Cr_2 11 °C [−16.0 kJ/mol] SmA 57 °C [−4.7 kJ/mol] I. ^1H NMR (300 MHz, $CDCl_3$): δ = 0.86–0.90 (m, 3H, CH_3), 1.23–1.45 (m, 10H, CH_2), 1.54–1.90 (m, 8H, CH_2), 2.97–3.02 (m, 2H, 2-CH_2), 3.33 (t, 2H, J = 6.6 Hz, CH_2N_3), 4.03 (t, 2H, J = 6.3 Hz, OCH_2), 6.99–7.04 (m, 2H, 3'-H, 5'-H), 7.47–7.51 (m, 2H, 2'-H, 6'-H), 8.83 (s, 2H, 4-H, 6-H) ppm. ^{13}C NMR (75 MHz, $CDCl_3$): δ = 14.1 (CH_3), 22.7, 23.4, 25.7, 28.7, 28.8, 28.9, 29.2, 29.5, 31.9 (CH_2), 39.2 (2-CH_2), 51.3 (CH_2N_3), 67.7 (OCH_2), 115.4 (C-3', C-5'), 127.9 (C-2', C-6'), 126.8, 130.8 (C-1', C-5), 154.5 (C-4, C-6), 159.5 (C-4'), 169.6 (C-2) ppm. FT-IR (ATR): ṽ = 2923 (m), 2853 (m), 2095 (s), 1607 (m), 1587 (m), 1608 (m), 1541 (m), 1517 (m), 1441 (s), 1244 (s), 1184 (m), 1025 (m), 996 (m), 834 (s) cm^{-1}. MS (ESI): m/z = 396.3 $[M + H]^+$. $C_{23}H_{33}N_5O$ (395.54): calc. C 69.84, H 8.41, N 17.71; found C 69.75, H 8.30, N 17.43.

5-[4-(4-cyanobutoxy)phenyl]-2-octylpyrimidine (7c)

Prepared according to general procedure (3). Experiment: 290 mg (0.59 mmol) bromide **3c**, 43.0 mg (0.65 mmol) KCN. Flash chromatography (PE/EtOAc, 5:1, v/v; $R_f = 0.17$) gave 209 mg (0.57 mmol, 97%) of **7c** as a colourless crystalline solid. Mp: 86 °C. ^1H NMR (500 MHz, $CDCl_3$): δ = 0.88 (t, 3H J = 6.9 Hz, CH_3), 1.22–1.45 (m, 10H, CH_2), 1.82–2.03 (m, 6H, CH_2), 2.47 (t, 2H, J = 7.0 Hz, CH_2CN), 2.96–3.03 (m, 2H, 2-CH_2), 4.07 (t, 2H, J = 6.0 Hz, OCH_2), 6.99–7.04 (m, 2H, 3'-H, 5'-H), 7.47–7.54 (m, 2H, 2'-H, 6'-H), 8.82 (s, 2H, 4-H, 6-H) ppm. ^{13}C NMR (125 MHz, $CDCl_3$): δ = 14.1 (CH_3), 17.0, 22.5, 22.7, 28.2, 28.9, 29.2, 29.5, 31.9 (CH_2), 39.2 (2-CH_2), 66.8 (OCH_2), 115.3 (C-3', C-5'), 119.4 (CN), 128.0 (C-2', C-6'), 127.2, 130.7 (C-1', C-5), 154.6 (C-4, C-6), 159.2 (C-4'), 169.8 (C-2) ppm. FT-IR (ATR): ṽ = 3037 (w), 2955 (m), 2917 (m), 2872 (m), 2848 (m), 1608 (m) 1588 (m), 1537 (m), 1517

(m), 1446 (s), 1393 (m), 1244 (s), 1183 (s), 1054 (m), 997 (m), 838 (s), 722 (m), 705 (m), 652 (m), 560 (m) cm^{-1}. MS (EI, 70eV): *m/z* (%) = 365.2 (59) $[M]^+$, 322.2 (8), 380.1 (29), 267.1 (100), 185.1 (11). HRMS (ESI): *m/z* $[M+H]^+$ calc. for $C_{23}H_{37}N_3O$: 366.2540; found: 366.2542. $C_{23}H_{31}N_3O$ (365.51): calc. C 75.58, H 8.55, N 11.50; found C 75.60, H 8.49, N 11.44.

5-[4-(5-cyanopropyloxy)phenyl]-2-octylpyrimidine (7d)

Prepared according to general procedure (3). Experiment: 173 mg (0.40 mmol) bromide **3d**, 52.0 mg (0.80 mmol) KCN. Flash chromatography (PE/EtOAc, 5:1, v/v; $R_f = 0.37$) gave 133 mg (0.35 mmol, 88%) of **7d** as a colourless crystalline solid. Mp: 67 °C. ^{1}H NMR (500 MHz, $CDCl_3$): $\delta = 0.88$ (t, 3H $J = 6.9$ Hz, CH_3), 1.21−1.44 (m, 10H, CH_2), 1.64–1.92 (m, 6H, CH_2), 2.40 (t, 2H, J = 7.0 Hz, CH_2CN), 2.95–3.01 (m, 2H, 2-CH_2), 4.03 (t, 2H, J = 6.3 Hz, OCH_2), 6.99–7.03 (m, 2H, 3'-H, 5'-H), 7.46–7.51 (m, 2H, 2'-H, 6'-H), 8.82 (s, 2H, 4-H, 6-H) ppm. ^{13}C NMR (125 MHz, $CDCl_3$): δ =14.1 (CH_3), 17.1, 22.7, 25.2, 25.4, 28.5, 28.9, 29.2, 29.5, 31.9 (CH_2), 39.2 (2-CH_2), 67.5 (OCH_2), 115.3 (C-3', C-5'), 119.5 (CN), 128.0 (C-2', C-6'), 127.02, 130.8 (C-1', C-5), 154.6 (C-4, C-6), 159.4 (C-4'), 169.7 (C-2) ppm. FT-IR (ATR): $\tilde{\nu}$ = 2954 (m), 2917 (m), 2872 (m), 2849 (m), 1607 (m) 1588 (m), 1537 (m), 1517 (m), 1446 (s), 1391 (m), 1234 (s), 1183 (s), 1051 (m), 993 (m), 838 (s), 722 (m), 704 (m), 653 (m), 561 (m) cm^{-1}. MS (APCI): *m/z* = 380.3 $[M]^+$, 285.2, 256.2, 186.1. HRMS (ESI): *m/z* $[M+H]^+$ calc. for $C_{24}H_{34}N_3O$: 380.2696; found: 380.2708. $C_{24}H_{33}N_3O$ (379.54): calc. C 75.95, H 8.76, N 11.07; found C 76.16, H 8.82, N 10.93.

5-[4-(3-cyanopropoxy)phenyl]-2-octylpyrimidine (7b)

A solution of 5-(4-hydroxyphenyl)-2-octylpyrimidine **8** (568 mg, 2.00 mmol) in 2.0 mL DMF was added drop wise to a suspension of NaH (40wt% in mineral oil, 52.0 mg, 2.20 mmol) and 4 mL abs.DMF at 0 °C. After stirring for 40 min at 0 °C, 1-bromo-3-propanenitrile 480 µL (71.0 mg, 48.0 mmol) was added and the reaction mixture was stirred 12 h at room temperature, followed by quenching with 10 mL H_2O and 20 mL CH_2Cl_2. The aqueous layer

was extracted with CH_2Cl_2 (3 × 10 mL), dried (Na_2SO_4) and the solvents were evaporated. The crude product was purified by flash chromatography (PE/EtOAc, 4:1, v/v) to give 229 mg (0.65 mmol, 33%) of **7b** as a colourless crystalline solid.

PE/EtOAc, 4:1, v/v; $R_f = 0.17$. Mp: 77 °C. ^{1}H NMR (500 MHz, $CDCl_3$): δ = 0.86–0.89 (m, 3H, CH_3), 1.22–1.46 (m, 10H, CH_2), 1.82–1.90 (m, 2H, CH_2), 2.15–2.22 (m, 2H, CH_2), 2.63 (t, 2H, 2H, J = 7.0 Hz, CH_2CN), 2.98–3.04 (m, 2H, 2-CH_2), 4.14 (t, 2H, J = 5.8 Hz, OCH_2), 7.00–7.05 (m, 2H, 3'-H, 5'-H), 7.48–7.53 (m, 2H, 2'-H, 6'-H), 8.84 (s, 2H, 4-H, 6-H) ppm. ^{13}C NMR (125 MHz, $CDCl_3$): δ = 14.1 (CH_3), 22.7, 25.4, 28.8, 29.2, 29.4, 31.9 (CH_2), 39.0 (2-CH_2), 65.5 (OCH_2), 115.4 (C-3', C-5'), 119.0 (CN), 128.0 (C-2', C-6'), 127.4, 130.8 (C-1', C-5), 154.5 (C-4, C-6), 159.0 (C-4'), 169.6 (C-2) ppm. FT-IR (ATR): $\tilde{\nu}$ = 3038 (w), 2955 (m), 2917 (m), 2871 (m), 2848 (m), 1607 (m) 1587 (m), 1537 (m), 1517 (m), 1445 (s), 1390 (m), 1250 (s), 1182 (s), 1049 (m), 934 (m), 837 (s), 827 (s), 722 (m), 705 (m), 616 (m), 559 (m) cm^{-1}. MS (EI, 70eV): m/z (%) = 351.2 (64) $[M]^+$, 308.1 (7), 266.1 (27), 253.1 (100), 185.0 (13). HRMS (ESI): m/z $[M+H]^+$ calc. for $C_{22}H_{30}N_3O$: 352.2383; found: 352.2385. $C_{22}H_{29}N_3O$ (351.49): calc. C 75.18, H 8.32, N 11.96; found C 75.09, H 8.28, N 11.82.

Polarizing optical microscopy

The textures of compounds **3a–d**, **4a–c,e**, **6b–d** and **9** are similar to those which are shown in Figure2, 4, 6 (results and discussion).

X-ray diffraction data

X-ray diffraction studies confirmed the mesophase structures of the examined samples (Table 4). In the wide angle region, broad scattering halos were observed indicating fluid structures for all examined substances. In the small angle region, for most of the samples one sharp reflection could be observed, indicating the long-range 1D-translational smectic order of the systems. Only for **4b**, a second order reflection of the smectic layer peak was seen.

Table 4: X-ray diffraction data for selected compounds.

Compound	Mesophase	*d* spacing [Å] obsd	Miller indices
**3b**	Sm at 42 °C	25.2	(001)
**3c**	Sm at 54 °C	26.1	(001)
**4b**	Sm at 43 °C	23.5	(001)
		12.0	(002)
**4e**	Sm at 50 °C	28.2	(001)
**6b**	Sm at 38 °C	24.9	(001)
**6d**	Sm at 45 °C	27.1	(001)
**6e**	Sm at 40 °C	29.0	(001)

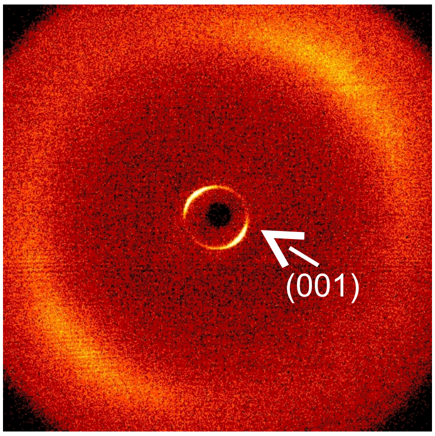

Figure 10: X-ray diffraction pattern (WAXS): a spontaneously aligned domain of SmA of the bromo-substituted compound **3c** at 54 °C (upon cooling from the isotropic phase).

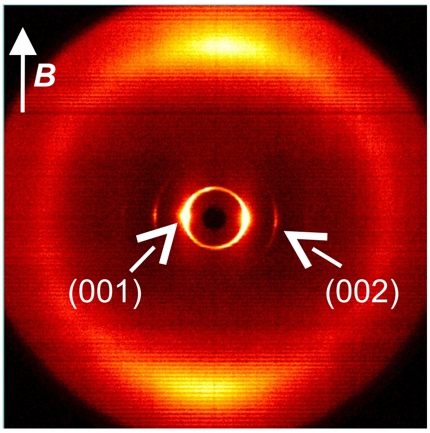

Figure 11: X-ray diffraction pattern (WAXS) of the SmA phase of the chloro-substituted compound **4b** at 43 °C (upon cooling from the isotropic phase and aligned in the magnetic field *B*).

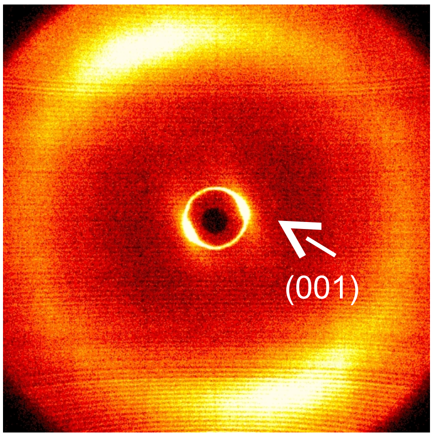

Figure 12: X-ray diffraction pattern (WAXS): a spontaneously aligned domain of SmA of the azido-substituted compound **6d** at 45 °C (upon cooling from the isotropic phase).
